# Functional Characterization of the *Stipa purpurea* *P5CS* Gene under Drought Stress Conditions

**DOI:** 10.3390/ijms22179599

**Published:** 2021-09-04

**Authors:** Danni Yang, Ruize Ni, Shihai Yang, Yanan Pu, Min Qian, Yunqiang Yang, Yongping Yang

**Affiliations:** 1 Plant Germplasm and Genomics Center, Kunming Institute of Botany, Chinese Academy of Sciences, Kunming 650201, China; yangdanni@mail.kib.ac.cn (D.Y.); niruize19@mails.ucas.ac.cn (R.N.); yang-shihai@163.com (S.Y.); puyanan94@163.com (Y.P.); qianmin181@163.com (M.Q.); 2 Institute of Tibetan Plateau Research at Kunming, Kunming Institute of Botany, Chinese Academy of Sciences, Kunming 650201, China; 3 Key Laboratory of Tibetan Environment Changes and Land Surface Processes, Institute of Tibetan Plateau Research, Chinese Academy of Sciences, Beijing 100085, China; 4 University of Chinese Academy of Sciences, Beijing 100049, China; 5 Yunnan Population and Family Planning Science and Technology Research Institute, Kunming 650021, China

**Keywords:** *Stipa purpurea*, *P5CS*, proline, drought tolerance

## Abstract

Free proline has multiple functions in plant cells, such as regulating osmotic potential and protecting both proteins and cell membranes. The expression of Δ1-Pyrroline-5-carboxylate synthase (P5CS), a key enzyme in the proline biosynthetic pathway, increases under drought, salt and cold stress conditions, causing plant cells to accumulate large amounts of proline. In this study, we cloned and identified the *P5CS* gene from *Stipa purpurea*, which has a full-length of 2196 bp and encodes 731 amino acids. A subcellular localization analysis indicated that SpP5CS localized to the cytoplasm. The ectopic overexpression of *SpP5CS* in *Arabidopsis thaliana* resulted in higher proline contents, longer roots, higher survival rates and less membrane damage under drought stress conditions compared with wild-type controls. *SpP5CS*-overexpressing *A. thaliana* was more resistant to drought stress than the wild type, whereas the deletion mutant *sp5cs* was less resistant to drought stress. Thus, *SpP5CS* may be a potential candidate target gene for increasing plant resistance to drought stress.

## 1. Introduction

Almost half of the agricultural area in the world is at risk of drought, which has become the most important problem in global agriculture, seriously affecting crop yields [1]. Increasing crop yields under drought conditions is a major goal of plant breeding, and physiological and molecular mechanisms of plant drought resistance have been studied to develop targets for molecular breeding. Drought can cause plant cells to produce excess reactive oxygen species (ROS) and free radicals. The accumulation of ROS and free radicals leads to membrane lipid peroxidation and subcellular structure damage, which destroys the integrity of plant cell membranes [2]. Water is an important solvent and reaction substrate in cells, and many biological macromolecules become permanently deformed and functionless after dehydration. To survive, plants under drought stress conditions accumulate some low molecular weight metabolites called osmolytes, including proline, glycine betaine, mannitol and quaternary amines. These osmolytes act as solutes to reduce the water potential in cells to improve the water absorption or water retention capacity of the cell while maintaining the cell volume and protecting the cell membrane [3].

The amino acid proline, as a common osmolyte, has many effects on plant cells under drought conditions. Under osmotic stress conditions, large quantities of proline accumulate in plant cells and the proline accumulation level varies among species, with levels in some species increasing 100-fold compared with the levels under normal conditions [4]. For example, sunflower (*Helianthus annus*) seeds accumulate more proline under drought or salt damage [5]. The accumulation of proline also responds to the induction of abscisic acid (ABA) and polyamines. The proline content of wheat increased significantly after treatment with exogenous ABA or polyamines [6,7]. The increase in free proline reduces the intracellular osmotic potential and maintains the intracellular water content. Under osmotic stress, proline can increase the activity of antioxidant enzymes [8]. Proline also acts as a molecular chaperone to retain and stabilize protein three-dimensional structures, which are required to maintain their normal functions [4]. A high proline level in a cell reduces the damage caused by free radicals, scavenges ROS and balances the cytoplasmic pH and redox states, thereby maintaining the basic metabolism of the cell [9,10]. Proline also stabilizes the internal membrane and helps maintain the integrity of cell structures and functions. Finally, proline aids in storing carbon and nitrogen [11].

Δ1-Pyrroline-5-carboxylate synthase (P5CS, E.C. 2.7.2.11, E.C. 1.2.1.41) is a key enzyme in the proline biosynthetic pathway, which regulates the proline contents in plant cells and improves resistance to osmotic stress. The major precursor of proline synthesis under adverse conditions is glutamic acid, which is catalyzed by P5CS and Δ1-Pyrroline-5-carboxylate reductase into proline [12]. Under stress conditions, proline accumulations in plant cells are mainly regulated by the enzyme P5CS. Studies in *Hordeum vulgare*, *Oryza sativa* and other plants have shown that the *P5CS* expression and activity levels are upregulated under drought and salt stress conditions, thus increasing the proline content [13,14,15,16]. As a rate-limiting enzyme in the proline biosynthetic pathway, P5CS regulates proline biosynthesis through feedback inhibition and transcription regulation [17]. Most plants have two *P5CS* isoforms, such as *Brassica napus*, *Phaseolus vulgaris* and *O. sativa* [18,19,20]. Recent studies have found three *P5CS* genes in *Lilium regale* and *Medicago truncatula* [21,22]. The transcriptional regulation mechanism of different isoforms of *P5CS* genes varies from species to species. In *O. sativa*, *OsP5CS1* is a housekeeping gene, and *OsP5CS2* responds to stress [20]. In *B. napus*, *BnP5CS1* and *BnP5CS2* are both induced by salt, ABA [18]. In *M. truncatula*, the expression levels of *MtP5CS2* and *MtP5CS3* increase under salt and drought stress, while the expression of *MtP5CS1* is constitutive [22]. From the overexpression of *PvP5CS1* and *PvP5CS2* from *P. vulgaris* in *Arabidopsis thaliana*, both genes increased the proline content and salt tolerance, but *PvP5CS2* had a stronger ability to synthesize proline under salt stress [19]. The overexpression of *P5CS* increased the tolerance to drought and salt stress in transgenic rice, tobacco and wheat by increasing the proline content [23,24,25]. The overexpression of *A. thaliana P5CS* in tobacco increases the transgenic tobacco’s proline content and enhances its resistance to osmotic stress compared with the controls. The *P5CS* gene from *Vigna aconitifolia*, under the control of the abscisic acid-inducible promoter complex, enhances the salt stress tolerance of transgenic sugarcane [26].

*Stipa purpurea* is a constructive species of alpine grass found on the Qinghai—Tibet Plateau. It readily survives in extremely arid areas and has a strong drought tolerance. Additionally, *S. purpurea* plays an important role in protecting the soil and is used as forage [27]. In this study, we cloned the *P5CS* gene from *S. purpurea* and measured the expression levels of this gene under soil drought, cold and salt conditions. Additionally, *SpP5CS* overexpression in *A. thaliana* verified its function in drought stress. An analysis of several phenotypes and physiological indicators showed that the ectopic expression of *SpP5CS* in *A. thaliana* leads to greater stress resistance compared with the WT. Thus, *SpP5CS* may be an important gene in the drought resistance mechanism of *S. purpurea*.

## 2. Results

### 2.1. Cloning and Structural Analysis of SpP5CS

**Figure 1:**
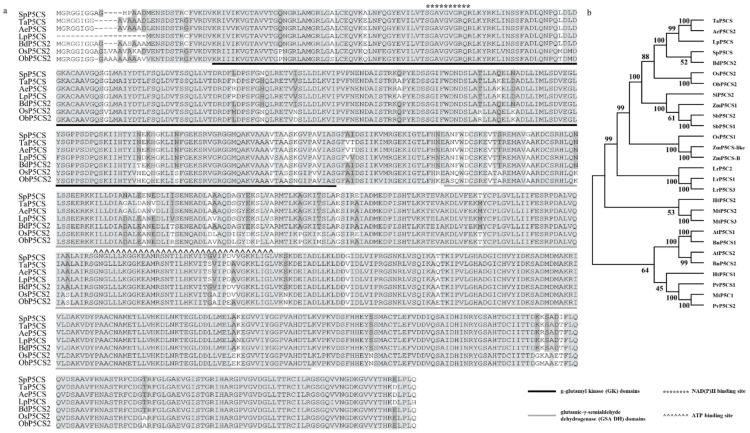
Sequences analysis of *SpP5CS*. (**a**) Multiple sequence alignment of *SpP5CS* and homologous proteins from other species. Legend shows the γ-glutamyl kinase (GK; 30–275 bp) and glutamic-γ-semialdehyde dehydrogenase (GSA DH; 299–600 bp) domains; (**b**) A phylogenetic tree constructed using SpP5CS and P5CS proteins from other plant species. *BdP5CS2* (*B. distachyon*, XP_003564608.1), *LpP5CS* (*L. perenne*, AGQ04179.1), *TaP5CS* (*T. aestivum*:KAF7024610.1), *AeP5CS2* (*A. tauschii*, XP_020198194.1), *OsP5CS1* (*O. sativa*, O04226.2), *OsP5CS2* (*O. sativa*, BAB64280.1), *ObP5CS2* (*O. brachyantha*, XP_006645010.2), *ZmP5CS1* (*Z. mays*, AQK99427.1), *ZmP5CSB*(*Z. mays*, AQK86404.1), *ZmP5CSB-like* (*Z. mays*, NP_001339256), *SbP5CS1* (*S. bicolor*, ACU65226.1), *SbP5CS2* (*S. bicolor*, ACU65227.1), *AtP5CS1* (*A. thaliana*, P54887.1), *AtP5CS2* (*A. thaliana*, P54888.1), *HtP5CS1* (*H. tuberosus*, AHJ08569.1), *HtP5CS2* (*H. tuberosus*,AHJ08570.1), *LrP5CS1* (*L. regale*, KT972374), *LrP5CS2* (*L. regale*, KT972375), *LrP5CS3* (*L. regale*, KT972376), *MtP5CS1* (*M. truncatula*, CAC82184.1), *MtP5CS2* (*M. truncatula*, AET87351.1), *MtP5CS3* (*M. truncatula*, AET35478.1), *PvP5CS1* (*P. vulgaris*, ABY61079.1), *PvP5CS2* (*P. vulgaris*, ABY89287.1)*, BnP5CS1* (*B. napus*, AF314811), *BnP5CS2* (*B. napus*, AF314812) and *SiP5CS2* (*S. italica*, XP_004970573.1).

Based on the EST sequence obtained by transcriptome sequencing, we used RACE (rapid amplification of cDNA ends) technology to obtain the CDS sequence of *SpP5CS*. The *SpP5CS* (GenBank: MZ710734) contains an open reading frame of 2196 bp that encodes 731 amino acids. The molecular weight of the SpP5CS protein is 78.95 kDa, and the theoretical isoelectric point is 5.95. The SMART protein structural analysis of SpP5CS revealed two functional domains: γ-glutamyl kinase (GK) and glutamic-γ-semialdehyde dehydrogenase (GSA DH) (Figure 1a). To analyze the relationship between *SpP5CS* and *P5CS* genes from other species, we extracted the *P5CS* gene sequences of *Brachypodium distachyon*, *Lolium perenne*, *Triticum aestivum*, *Aegilops tauschii*, *O. sativa*, *Oryza brachyantha*, *Zea mays*, *Sorghum bicolor, A. thaliana, Helianthus tuberosus, L. regale, M. truncatula, P. vulgaris*, *B. napus* and *Setaria italica* through the BLAST tool of NCBI (https://blast.ncbi.nlm.nih.gov/Blast.cgi, accessed on 6 August 2021). A phylogenetic tree was constructed using the BLAST tool of MEGA X and the neighbor-joining method. *SpP5CS* is highly similar to *P5CS* from *B. distachyon* (Figure 1b). The results indicate that *SpP5CS* belongs to the *P5CS2* isoform.

### 2.2. The Expression of SpP5CS and the Proline Content Analysis

**Figure 2:**
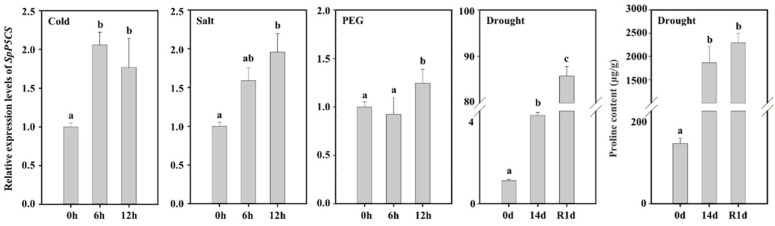
qPCR analysis of the expression levels of *SpP5CS* under cold, salt, drought and soil drought stress conditions. The experiment has three biological replicates, and the error bar denotes the standard deviation. The data are analyzed by one-way ANOVA (Tukey’s test). Different letters indicate significant differences among the relative expression levels of the genes at *p* < 0.05.

qRT-PCR was used to detect the expression of the *SpP5CS* in *S. purpurea* leaves after exposure to different treatments. After growing for 2 weeks, *S. purpurea* was exposed to different abiotic stress conditions (cold, salt, PEG and soil drought) for different times. After 6 h of the cold-stress treatment at 4 °C, *SpP5CS* expression increased to two times that of the control, but after 12 h of continuous treatment, the expression level decreased to 1.7 times that of the control. Additionally, after being treated with 150-mM NaCl for 6 h, *SpP5CS* expression increased to 1.6 times that of the control, and it continued to slowly increase to 1.9 times that of the control by 12 h. After 12 h of treatment with 30% PEG 6000, the expression of *SpP5CS* increased slightly compared to the control. After 14 d of soil drought, the expression of *SpP5CS* increased 3.8 times compared with the control, and after 1 d of rehydration, the expression increased significantly, to 85.7 times that of the control (Figure 2). The *SpP5CS* gene was upregulated under the cold, salt and PEG stress conditions. The expression of the *SpP5CS* gene was strongly and significantly upregulated during soil drought and rehydration treatment. Furthermore, we determined the proline content of *S. purpurea* leaves corresponding to 0 d and 14 d of soil drought and 1 d of rehydration. The proline content increased 14.4 times in comparison to the control after 14 d of drought treatment and 17.6 times after 1 day of rehydration. The results of the expression level analysis of the *SpP5CS* gene are consistent with the results of the proline content. These results indicate that *SpP5CS* actively responds to soil drought and increases the proline content of *S. purpurea* under drought stress.

### 2.3. Identification of Ectopically Expressed 35S:SpP5CS-GFP Transgenic Plants

**Figure 3:**
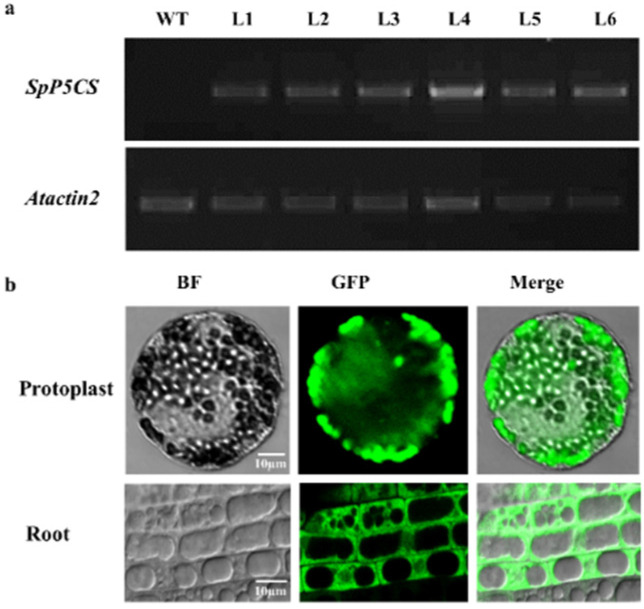
RT-PCR identification of *SpP5CS* and the subcellular localization of the 35S:*SpP5CS*-GFP fusion protein. (**a**) The RT-PCR identification of lines 1-6 of OE-SpP5CS *A. thaliana*; (**b**) Subcellular localization of the 35S:SpP5CS-GFP fusion protein in the root tips and leaf mesophyll protoplasts of transgenic *A. thaliana*.

To further explore *SpP5CS*’s functions, we cloned the gene and transformed it into *A. thaliana*, with the expression being driven by the 35S Cauliflower mosaic virus promoter. The primers SpP5CS-GFP-F and SpP5CS-GFP-R were used to successfully identify the transgenic plants (Figure 3a). We received six transgenic lines, and the transformation efficiency was about 0.1%. We observed the SpP5CS-GFP fusion protein in the cytoplasm from transgenic *A. thaliana* using a laser confocal microscope (Figure 3b).

### 2.4. SpP5CS Overexpression Enhances Drought Tolerance

**Figure 4:**
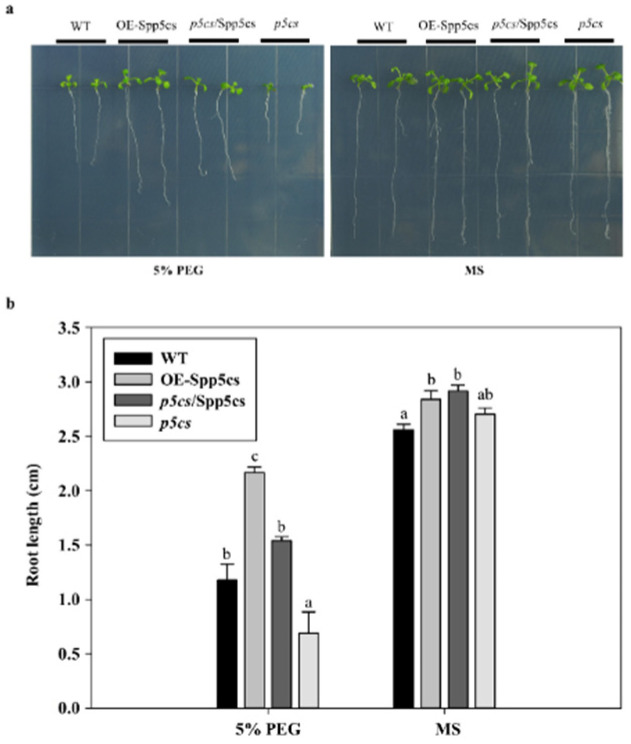
Root length analysis of WT, OE-SpP5CS, *p5cs*/SpP5CS-complemented and *p5cs* mutant *A. thaliana* seedlings in response to drought stress. (**a**) Growth of plants under drought stress conditions. Seeds were grown on the 1/2 MS medium and 1/2 MS medium supplemented with 5% PEG 6000 for 10 d; (**b**) Root lengths of WT, OE-SpP5CS, *p5cs/SpP5CS*-complemented and *p5cs* mutant plants grown under normal and drought stress conditions. Data were obtained from three independent experiments. Each column represents the mean ± SD, and the error bar denotes the standard deviation. Different letters indicate significant differences in the root length (one way ANOVA, Tukey’s test, *p* < 0.05).

To analyze whether *SpP5CS* expression enhances the plant’s drought resistance function, the *SpP5CS* gene was first transformed into a *P5CS* deletion mutant to obtain a *p5cs*/SpP5CS complemented plant. Then, the WT, mutant, *p5cs*/SpP5CS-complemented and OE-SpP5CS *A. thaliana* seeds were cultured on 1/2 Murashige and Skoog (MS) medium supplemented with 5% PEG 6000 and ordinary 1/2 MS medium, and the phenotypes were observed. On 1/2 MS medium containing 5% PEG 6000, the average root length of the OE-SpP5CS plants was 2.17 cm, and the average root length of a mutant plants was 0.69 cm. The root lengths of OE-SpP5CS plants under drought conditions were significantly longer than those of the other types of plants, whereas the mutant plants themselves were significantly shorter than the other types of plants (Figure 4a,b). The average root lengths were 1.18 cm and 1.54 cm, respectively, on 1/2 MS medium, with no significant differences among the root lengths of the various plants.

**Figure 5:**
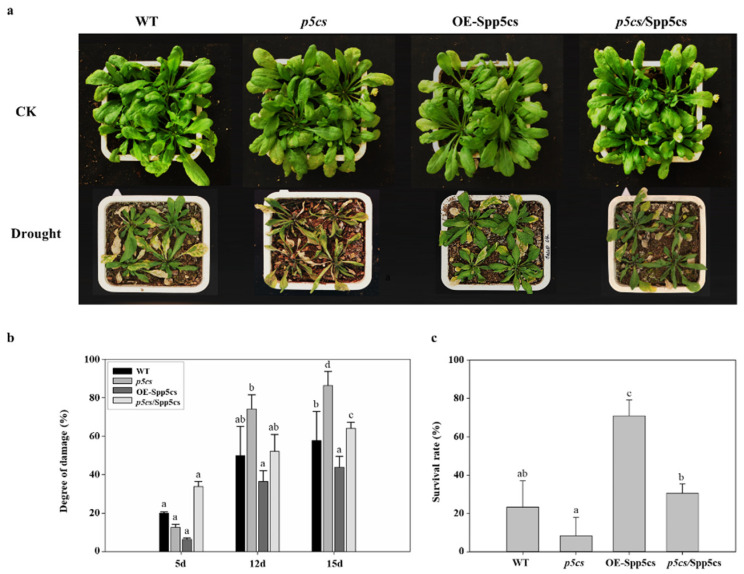
Phenotypic and membrane damage analyses of WT, OE-SpP5CS, *p5cs/SpP5CS*-complemented and *p5cs* mutant *A. thaliana* plants. (**a**) Phenotypes of WT, OE-SpP5CS, *p5cs/SpP5CS*-complemented and *p5cs* mutant plants in response to 12 days of soil drought stress. CK is the control group under the condition of normal irrigation; (**b**) Comparison of the degree of membrane damage among WT, SpP5CS, *p5cs/SpP5CS*-complemented and *p5cs* mutant plants in response to soil drought stress; (**c**) Comparison of the survival rates among WT, SpP5CS, *p5cs/SpP5CS*-complemented and *p5cs* mutant plants after 12 days of soil drought stress. The soil drought treatment adopts continuous withholding water. The experiment has three biological and technical replicates. The data are expressed as the mean ± SD from three independent experiments. Different letters indicate significant differences in the degree of damage or survival rate (one-way ANOVA, Tukey’s test, *p* < 0.05).

To further identify the functions of *SpP5CS* in improving plant drought resistance, WT, mutant, *p5cs*/SpP5CS-complemented and OE-SpP5CS *A. thaliana* seedlings were planted in square plastic pots and subjected to the soil drought treatment. After 15 d of the drought treatment, the growth status of OE-SpP5CS *A. thaliana* was the best among all the plant types (Figure 5a). Then, their survival rates and electrical conductivity levels were measured. As shown in Figure 5c, after 5 d of drought treatment, the survival rate of OE-SpP5CS *A. thaliana* was 87.4%, and the plants showed the lowest degree of damage at 6.4%, while the survival rate of the mutant *A. thaliana* was 66.1%, and the plants showed the greatest degree of damage at 33%. After 12 d of drought treatment, the survival rate of OE-SpP5CS *A. thaliana* was 63.6%, and the damage degree was 36.4%, while the survival rate of the mutant *A. thaliana* was 25.8%, and the damage degree was 74.2%. After 15 d of drought treatment, the survival rate of the OE-SpP5CS *A. thaliana* was 56.1%, and the damage degree was 43.9%, while the survival rate of the mutant *A. thaliana* was 13.7%, and the damage degree was 86.3% (Figure 5b,c). The survival rates and degrees of damage for the WT and *p5cs*/SpP5CS-complemented *A. thaliana* were at intermediate levels, with little differences between the two lines. Therefore, the overexpression of *SpP5CS* improved the drought resistance of transgenic *A. thaliana*.

### 2.5. Variation in the Proline Content

**Figure 6:**
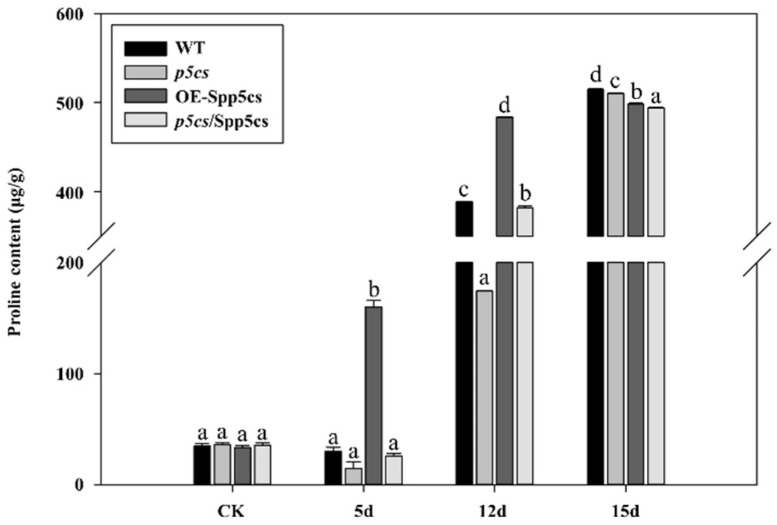
Proline contents of WT, OE-SpP5CS, *p5cs/SpP5CS*-complemented and *p5cs* mutant plants after the soil drought treatment. The soil drought treatment adopts continuous withholding water. CK is the control group under the condition of normal irrigation. The experiment has three biological and technical replicates. Data are the mean values ± SD obtained from three independent experiments (error bars = SD, *n* = 15). Different letters indicate significant differences in the proline content (one-way ANOVA, Tukey’s test, *p* < 0.05).

To investigate the effects of *SpP5CS*, the proline content was determined for WT, *p5cs* mutant, *p5cs*/SpP5CS-complemented and OE-SpP5CS *A. thaliana* using the acidic-ninhydrin assay. After 5 d of drought treatment, the proline content of the OE-SpP5CS *A. thaliana* was significantly higher than the other plant types and more than five times that of the WT. At this time, the proline content of the mutant was the lowest among all the plants at approximately half the WT value. After 12 d of drought treatment, the proline content of the OE-SpP5CS *A. thaliana* was still the highest, but the differences compared with the WT decreased. The mutant still had the lowest content. After 15 d of drought treatment, the proline contents of the plants were nearly the same (Figure 6). These results indicate that the *SpP5CS* gene improves the drought resistance of plants by increasing their proline content.

## 3. Discussion

### 3.1. Molecular Characterization of SpP5CS

As an important gene involved in proline synthesis, *P5CS* has been reported in *O. sativa*, *P. vulgaris*, *A. thaliana* and *S. bicolor*, and it contains the conserved domains GK and GSA DH [28,29]. In this study, we cloned the full-length CDS sequence of *SpP5CS* gene from *S. purpurea*. Then, we performed multiple sequence alignment and phylogenetic analysis of the P5CS protein of *S. purpurea* and other species. We found that they have similar structural domains (GSA DH and GK), and SpP5CS and P5CS2 of multiple species are in the same branch. Due to their structural similarity, SpP5CS may have functions similar to P5CS2 in other species. According to previous reports, the research of *P5CS2* mainly focused on growth and development, drought resistance and salt resistance [18,19,20,21,22,30]. We speculate that SpP5CS has a similar effect. Previous subcellular localization studies have shown that P5CS2 are localized in the cytoplasm [30]. The subcellular localization results show that SpP5CS is localized in the cytoplasm, which is consistent with the previous report.

### 3.2. Expression Analysis of SpP5CS

The gene expression analysis shows differences in the gene expressions under different environmental conditions, which helps predict the gene function. The *P5CS* gene responds to osmotic stress and increases the proline content of plants to improve osmotic resistance. In *S. italica*, *SiP5CS2* is remarkably upregulated after 9 d of drought treatment [31]. After 10 d of salt stress, the expression of *Opuntia streptacantha P5CS* increases [32]. Salt-tolerant varieties of *O. sativa* have more *OsP5CS* mRNA transcripts and proline under high-salt than normal conditions [14]. The expression of *Setaria viridis P5CS2* is upregulated after 6 and 10 d of an osmotic stress treatment in the form of an external PEG 8000 application [33]. In addition to the response to osmotic stress, the *P5CS* gene is also upregulated during cold stress. After 24 h of a cold treatment at 4 °C, the transcription level of the *P5CS* gene in *L. perenne* is significantly upregulated [34]. The expression of *SpP5CS* was significantly upregulated under soil drought, cold and salt stress, which was consistent with the results of the other species. According to reports, different isoforms of *P5CS* genes are induced by different stresses. In Sorghum bicolor, the transcription levels of *SbP5CS1* and *SbP5CS2* are both upregulated under drought and salt stress. Meanwhile, the transcription level of *SbP5CS2* increased faster and more obviously than that of *SbP5CS2* under the induction of methyl jasmonic acid [29]. In our study, *SpP5CS* strongly responds to soil drought, which may be the result of *S. purpurea* adapted to arid environment for a long time. In addition, the proline content of *S. purpurea* increased significantly after 14 days of soil drought and 1 day after rehydration. We speculate that *S. purpurea* accumulates proline by increasing the expression of the *SpP5CS* gene to improve the drought resistance after drought stress.

### 3.3. Potential Mechanism of SpP5CS for Improving Plant Resistance to Drought Stresses

There are various drought resistance mechanisms in plants, among which, an increasing root length and increasing osmotic pressure regulatory substances are very common. As a key enzyme in the proline biosynthesis pathway, P5CS also enhances root growth, and it plays an important role in plant resistance to drought. The electrical conductivity of cell leakage indirectly evaluates the degree of cell membrane damage [35]. OE-SpP5CS *A. thaliana* showed the lowest degree of membrane damage compared with WT. *P5CS* transgenic rice increase their proline contents under water shortage conditions and have increased fresh shoot weights, by 50–95%, after PEG treatments [23]. The overexpression of *SpP5CS* in *A. thaliana* increases the proline content to combat drought stress. Combining the results of the heterologous expression in *A. thaliana* and qPCR in *S. purpurea*, *SpP5CS* can enhance the drought resistance of plants by increasing the proline production.

*S. purpurea* is widely distributed in the precipitation gradient of the Qinghai-Tibet Plateau, and it can still survive in extremely arid areas. Previous studies have revealed that *S. purpurea* in arid regions have a higher content of proline than *S. purpurea* in humid regions, and a transcriptome analysis has shown that the *P5CS* gene is upregulated at a higher level in arid regions [36]. The proteomic analysis also suggests that the P5CS protein in *S. purpurea* from more arid areas has a high level of upregulation [37]. Furthermore, we verified the single gene function of *SpP5CS* to show that *SpP5CS* improves the drought resistance of plants. These studies all indicate that the upregulation of *SpP5CS* gene leads to an increase in the proline content to enhance osmotic regulation, which is an important part of the drought-resistant mechanism of *S. purpurea*.

## 4. Materials and Methods

### 4.1. Plant Materials

Mature *S. purpurea* seeds were collected from Nyima County, Tibet (32°00′05″ N, 86°50′54″ E), China. The seeds were planted in square plastic pots (10 cm × 10 cm × 8 cm) containing nutrient soil (Pindstrup Mosebrug A/S, Ryomgaard, DK) and placed in the dark to germinate. After that, the seedlings were moved into a greenhouse and grown for about 2 weeks (trefoil stage) for cold and soil drought treatments. After 0, 6, and 12 h of cold treatment in a plant incubator (SAFU EXPERIMENTAL APPARATUB TECHNOLOGY, China) at 4 °C, the second and third leaves of *S. purpurea* were collected. For the soil drought treatment, the second and third leaves of *S. purpurea* were selected after continuously withholding water for 0 d and 14 d and rehydration (fully watered) for 1 day. For salt and PEG treatment, *S. purpurea* seeds were soaked in water overnight and germinated on moist filter paper in the dark. After germination, the seedlings were transplanted into a plastic pot (10 cm × 10 cm × 8 cm) containing the same amount of vermiculite and 1/2 liquid MS in a greenhouse for about 2 weeks (trefoil stage). After that, the 1/2 MS was replaced with 1/2 MS containing 150-mM NaCl or 30% polyethyleneglycol 6000 (PEG 6000). After 0, 6 and 12 h of salt or PEG treatment, the second and third leaves of *S. purpurea* were collected [38,39]. The samples were frozen in liquid nitrogen and stored at −80 °C for subsequent RNA isolation. *A. thaliana* plants (ecotype: Columbia ecotype, Col-0) were cultured for ectopic expression experiments on 1/2 solid MS medium. After 10 days, the seedlings were transferred to nutrient soil. The *p5cs* mutant (SALK_045245) was purchased from The Arabidopsis Information Resource (https://www.arabidopsis.org/, accessed on 11 June 2016). All the plants were grown in a greenhouse at 21 °C, with 75–80% relative humidity and a light intensity of 200-μmol photons m^−2^s^−1^, under a 16-h light/8-h dark photoperiod. For untreated *A. thaliana* and *S. purpurea*, the normal irrigation situation was 30 mL of water every two days [37].

### 4.2. RNA Isolation and SpP5CS Gene Cloning

Total RNA was isolated from leaves of *S. purpurea* using an Eastep^®^ Super Total RNA Extraction Kit (Promega, Madison, WI, USA), and the DNA digested with DNase I. RNA was quantified using a NanoDrop1000 (NanoDrop Technologies, Wilmington, DE, USA), and the integrity was checked on a 0.8% agarose gel. Then, RNA (5 µg) was reverse-transcribed into first-strand cDNA using the GoScript™ Reverse Transcription System (Promega, Madison, WI, USA). We used RACE technology to obtain the full length of the *SpP5CS* sequence. Based on the transcriptome data (the Genome Sequence Archive in the BIG Data Center, Beijing Institute of Genomics (BIG), accession numbers CRA002018), the required EST fragments were screened out, and specific primers SpP5CS-RACE-1 (5′-TCTCGCGAAGCTGGTTCCCAAAACT-3′) and SpP5CS-RACE-2 (5′-GGGCCTGTTGCACATGACACACTGAA-3′) were designed. Then we used SMART RACE cDNA kit (BD Biosciences Clontech, MountainView, CA, USA) to amplify the 5′and 3′ sequences of the target gene. Then specific primer SpP5CS-F (5′-ATGGGTAGAGGAGGGATCGGA-3′) and SpP5CS-R (5′-TCATTGCAACGGAAGCTCCCTGTGG-3′) were used to obtain the full-length CDS sequence of *SpP5CS*. We constructed the full length of *SpP5CS* into the pMD 18-T vector (Takara Bio, Kusatsu, Japan), and applied Sanger sequencing for paired-end sequencing.

### 4.3. Sequence Analysis of the SpP5CS Gene

The full-length *SpP5CS* cDNA sequence was translated into a protein using DNAMAN (Lynnon Biosoft, San Ramon, CA, USA). The protein was analyzed using the online SMART tool (http://smart.embl-heidelberg.de/smart/show_motifs.pl, accessed on 5 October 2018) and ExPASy ProtParam tool (https://web.expasy.org/protparam/, accessed on 8 October 2018). The protein sequences of other species were obtained using the BLAST tool of NCBI and aligned using the Clustal X program [40]. Then, a maximum parsimony analysis was performed using MEGA X with the neighbor-joining method and the 1000 bootstrap test replicates [41].

### 4.4. Gene Expression Analysis

To quantify the *SpP5CS* expression levels under different abiotic stress conditions, the cDNAs of processed *S. purpurea* samples were used as templates for quantitative real-time (qRT)-PCR analyses. The ABI 7500 Fast Real-time PCR System (Thermo Fisher Scientific, Waltham, MA, USA) and FastStart Universal SYBR Green Master (Roche, Indianapolis, IN, USA) were used. The reaction parameters for thermal cycling were as follows: 95 °C for 10 min, followed by 40 cycles of 94 °C for 5 s and 60 °C for 15 s. The reaction system (total 20 μL) was as follows: cDNA template (dilute 5 times) 2 μL, nuclear-free water 6.8 μL, primer (10 μM) 0.6 μL, SYBR Green Master 10 μL. qRT-PCR was conducted in triplicate with different cDNAs synthesized from three biological replicates. The gene-specific primer SpP5CS-qPCR-F/-R (SpP5CS-qPCR-F: 5′-GGTGATTCTGGTCACCTCCG-3′, SpP5CS-qPCR-R: 5′-CCTTCCCATCCAGGTCCAAC-3′) was used to amplify *SpP5CS*, with *SpACT2* (F: 5′-CTGTGCCAATCTACGAGGGT-3′ and R: 5′-GAACCACCGATCCAGACACT-3′) as the internal control. The data were analyzed using the 2^−△△Ct^ method [42].

### 4.5. Generation and Screening of Transgenic A. thaliana Plants

The full-length SpP5CS CDS was inserted into the vector pRI 101 green fluorescent protein (GFP) and driven by the 35S Cauliflower mosaic virus promoter. Then, the recombinant plasmid was transformed into *Agrobacterium tumefaciens* stain GV3101 that was used to transform WT and *p5cs* mutant *A. thaliana* by the floral dip method [43]. The harvested seeds were grown on MS medium supplemented with 50-mg L^−1^ kanamycin to select for positive transgenic plants. The transgenic *A. thaliana* were examined by RT-PCR using *SpP5CS*-GFP-F (5′-GACCCCGGGGGTACCGGATCCATGGGTAGAGGAGGGATCGGA-3′) and *SpP5CS*-GFP-R (5′-TCAGAATTCGGATCCTCATTGCAACGGAAGCTCCCTGTGG-3′). The *A. thaliana* leaves of the same period were taken to extract RNA and reverse to obtain cDNA (according to the method in Section 4.2). *Atactin2* (NM_001338358) were used to normalize the *A. thaliana* samples. The PCR reaction was performed under following conditions: 25 cycles at 95 °C for 30 s, 55 °C for 30 s, 72 °C for 2 min.

### 4.6. Subcellular Localization of SpP5CS

The *SpP5CS*-overexpression (OE-SpP5CS) and WT *A. thaliana* lines were germinated on 1/2 MS medium until the root lengths were approximately 2 cm, and then, the roots were cut for subcellular localization. Leaf mesophyll protoplasts were isolated from ten-day-old cotyledons of OE-SpP5CS transgenic *A. thaliana*. The method of protoplast isolation was based on previous research [44]. A laser scanning confocal microscope (Olympus Optical, Tokyo, Japan) was used to observe the GFP expression.

### 4.7. Drought Stress, Survival Rate, and Root-Length Measurements

For drought tests, seeds of WT, *p5cs* mutant, *p5cs*/SpP5CS-complemented and OE-SpP5CS *A. thaliana* plants were sown on 1/2 MS medium and 1/2 MS medium supplemented with 5% PEG 6000. Root lengths were measured after 10 d. To determine the transgenic phenotypes under drought and normal irrigation conditions (30 mL of water every two days), WT, mutant, *p5cs/SpP5CS*-complemented and OE-SpP5CS *A. thaliana* seedlings were planted in plastic pots filled with nutrient soil and grown in a greenhouse under normal irrigation conditions. Four-week-old seedlings with about 17 true leaves were used for drought stress treatment. Drought conditions were simulated by withholding water for 5, 12 or 15 d. After each treatment, the plants were sampled, quickly frozen in liquid nitrogen and stored in a refrigerator at −80 °C for subsequent experiments. During the drought treatment process, the survival rate of *A. thaliana* at each drought stage was calculated.

### 4.8. Relative Electrical Conductivity Measurement

The relative conductivity of *A. thaliana* at different treatment periods was measured simultaneously. The fresh leaves (4th to 6th leaves) were weighed (about 0.3 g), cut into 0.5-mm lengths and placed in a tube containing 10 mL of distilled water. To extract the air between the cells, the leaves in the distilled water were pumped in a vacuum for 20 min. Then, the samples were placed at room temperature for 1 h, and the conductivity of the solution were measured (S1). The solution was then heated to 100 °C for 10 min, cooled to room temperature and the final electrical conductivity (S2) was measured. The relative electrical conductivity (REC) was calculated as follows: REC = S1/S2. The degree of damage was calculated as follows: (REC_TREATMENT_ − REC_CONTROL_)/(1 − REC_CONTROL_) × 100%. The measurement method was based on the previously described method [35,45].

### 4.9. Proline Content Measurement

The proline content was measured by a previously described method [46]. The sample was weighed (about 0.4 g) and put in a 10-mL centrifuge tube containing 5 mL of 3% (*w*/*v*) 5-sulfosalicylic acid dihydrate and boiled for 10 min. Two milliliters of boiling supernatant were reacted with 2 mL of glacial acetic acid and 2 mL of ninhydrin and boiled for 30 min. When the temperature of the reaction mixture reached room temperature, it was extracted with 4 mL of toluene, and the supernatant was measured at 520 nm. At the same time, the same method was used to determine the absorbance values of different standards of proline to construct a standard curve. Finally, the proline content was calculated according to the standard curve.

## 5. Conclusions

In this study, the *P5CS* gene cloned from *S. purpurea* had the same structure as the *P5CS* genes of other plant species. The expression level of *SpP5CS* was significantly upregulated under drought stress conditions, and SpP5CS localized to the cytoplasm. The overexpression of *SpP5CS* in *A. thaliana* increased the drought resistance and root lengths, and it reduced damage to the cell membrane. We speculate that, under drought stress conditions, the *SpP5CS* expression level increases, which further increases the proline contents of plant cells, thereby improving the drought resistance of the plants.

## Data Availability

Not applicable.
